# Relative value of sleep and sleep-related complaints in people without sleep disorders: mediating role of cognitive, emotional and behavioral factors

**DOI:** 10.1192/j.eurpsy.2022.2096

**Published:** 2022-09-01

**Authors:** E. Rasskazova

**Affiliations:** 1 Mental Health Research Center, Medical Psychology, Moscow, Russian Federation; 2 Moscow State University, Clinical Psychology, Moscow, Russian Federation

**Keywords:** sleep-related complaints, value of sleep, psychological factors

## Abstract

**Introduction:**

High prevalence of dysfunctional beliefs about sleep and poor sleep hygiene in population (Perlis et al., 2011, Riemann et al., 2017) allow suggesting (Rasskazova, Tkhostov, 2012) a socially determined low value of sleep relative to other activities and demands.

**Objectives:**

The aim was to reveal the role of the relative value of sleep and subjective quality of sleep in people without sleep disorders.

**Methods:**

172 participants 18-62 years old without diagnosed sleep disorders answered three items about their relative sleep value, filled Insomnia Severity Index, Dysfunctional Beliefs About Sleep Scale (Morin, 1993), Behavioral Factors of Sleep Disorders Scale (Rasskazova, 2020) and Hospital Anxiety and Depression Scale (Zigmond, Snaith, 1983)

**Results:**

56.3% -65.3% participants tend to neglect sleep for the sake of other activities in conflictual situation independent on gender and age. Sleep neglect is associated with poorer subjective sleep indirectly – through poor sleep hygiene, depressive emotions and postponement of the time to get up in the morning (β=.02-.09; 95%CI [.01-.17]). High value of healthy sleep is associated with poorer sleep quality if it leads to higher dysfunctional sleep beliefs and sleep rituals (indirect effects β=.04-.16; 95%CI [.01-.23]), but with better sleep quality if it leads to better sleep hygiene in the evening and less delay in getting up in the morning (β=-.04 - -.02; 95%CI [-.08-.00]).

**Conclusions:**

Relative value of sleep might play a different role in the sleep regulation depending on which long-term beliefs, emotions, and behaviors it provokes. Research is supported by the Russian Foundation for Basic Research, project No. 20-013-00740.

**Disclosure:**

Research is supported by the Russian Foundation for Basic Research, project No. 20-013-00740

